# Comparison of Riboflavin/Ultraviolet-A Cross-Linking in Porcine, Rabbit, and Human Sclera

**DOI:** 10.1155/2014/194204

**Published:** 2014-01-02

**Authors:** Yali Zhang, Zhiwei Li, Lei Liu, Xuguang Han, Xiaomin Zhao, Guoying Mu

**Affiliations:** ^1^Department of Ophthalmology, The Second People's Hospital of Jinan, 148 Jingyi Road, Jinan, Shandong 250001, China; ^2^Department of Ophthalmology, Shandong Provincial Hospital Affiliated to Shandong University, No. 324 Jingwuweiqi Road, Jinan, Shandong 250021, China; ^3^School of Nursing, Binzhou Medical University, No. 346 Guanhai Road, Laishan District, Yantai, Shandong 264003, China

## Abstract

*Purpose*. To compare the biomechanical properties of porcine, rabbit, and human sclera before and after riboflavin/ultraviolet-A (UVA) collagen cross-linking (CXL). *Methods*. Eight rabbits, 8 porcine eyeballs, and 8 human eyeballs were included. One rabbit eye and half of each bisected human and porcine eyeball were treated with riboflavin/UVA CXL. Untreated fellow rabbit eyes and eyeball halves served as controls. A 10 mm × 20 mm scleral band was harvested from each specimen. From this band, two 3.5 mm × 15.0 mm strips were prepared for biomechanical testing. The biomechanical parameters were ultimate stress, stress and Young's modulus. *Results*. Values of stress, and Young's modulus showed that human sclera was 4 times stiffer than porcine sclera and 3 times stiffer than rabbit sclera. In rabbit sclera, both the stress and Young's modulus were significantly increased by CXL (*P* < 0.05). In porcine sclera, only the ultimate stress was significantly increased by CXL (*P* < 0.05). The biomechanical properties of human sclera were not statistically affected by CXL (*P* > 0.05). *Conclusions*. Human sclera has higher biomechanical stiffness than porcine and rabbit sclera. With the same irradiation dose, riboflavin/UVA CXL increases the biomechanical stiffness of rabbit sclera but not porcine or human sclera.

## 1. Introduction

Myopia is a common ocular disorder and is traditionally subdivided into stationary and progressive myopia. Myopia progression is more common in teenagers, and Bullimore et al. [1] also found that myopia progression is common in adults. As the eyeball continues to lengthen, scleral thinning occurs, particularly at the posterior pole [2].

The sclera is not a static container of the eye but rather a dynamic tissue that responds to changes in ocular size and refraction [3]. Studies have shown that biomechanical parameters of the sclera are weaker in myopic human eyes than in age-matched nonmyopic eyes [4]. Therefore, techniques to strengthen the posterior sclera were developed in the hopes of halting myopic progression and subsequently decreasing visual loss. Posterior scleral reinforcement surgery is one such effective method, with evidence that it can control axial myopia [5]. Collagen cross-linking (CXL) is another method to strengthen the sclera and is divided into 2 types: chemical cross-linking (using glucose, ribose, glyceraldehyde, glutaraldehyde, aliphatic beta-nitro alcohol, etc.), and physical cross-linking (using rose bengal/white light, riboflavin/ultraviolet A light, riboflavin/blue light, etc.) [6–8]. Wollensak and Spoerl [6] studied CXL in human and porcine sclera and showed that chemical CXL strengthens the sclera to a greater extent than does physical CXL. Because treatment placement is easier to control with physical CXL, physical, rather than chemical, methods have been tested in experimental and clinical studies of progressive keratoconus [9, 10], iatrogenic keratectasia [11], corneal melting [12], and keratitis [13].

In 2004, Wollensak and Spoerl [6] compared the *in vitro* efficacy of various CXL to increase scleral biomechanical strength in human and porcine eyes. They found that riboflavin/UVA, glyceraldehyde, and glutaraldehyde CXL significantly stiffened both human and porcine sclera. Additionally, later *in vivo* studies with riboflavin/UVA CXL in rabbits also showed that the CXL treatment was effective in strengthening scleral tissue [14, 15], and the effect can be constant up to 8 months [15]. For ethical reasons, scleral CXL in live human tissue is limited to fresh enucleated eyes. Because of extremely limited availability, porcine and rabbit sclera are often used as models for human sclera tissue. The purpose of the current study was to compare the biomechanical properties of porcine, rabbit, and human sclera both before and after riboflavin/UVA CXL, in preparation for the research involving animal models of scleral CXL.

## 2. Materials and Methods

This study followed the principles of animal use and maintenance, as described in the Association for Research in Vision and Ophthalmology Statement for Use of Animals in Ophthalmic and Vision Research. The study also adhered to the tenet of the Declaration of Helsinki regarding the use of human tissues.

### 2.1. Specimen Preparation

Eight adult rabbits, weighing 2.0–2.5 kg, were included in this study. Rabbits were sacrificed before the CXL procedure with an overdose of sodium pentobarbital injected intravenously. Because rabbit eyes are relatively small, one eye from each rabbit was treated, while the other eye remained untreated and served as a control. Scleral bands were harvested following orbit enucleation. Eight adult porcine cadaver eyes were obtained within 12 hours of death from a local abattoir. Eight human eyes with a donor age of 28 to 43 years were retrieved within 12 hours of death from the Red Cross eye bank of Shandong province after the corneas had been removed. The donors were free of ocular disease, collagen disease, and diabetes mellitus. Both porcine and human eyeballs were bisected and one-half was treated with riboflavin/UVA CXL, while the other half served as a control.

**Figure 1:**
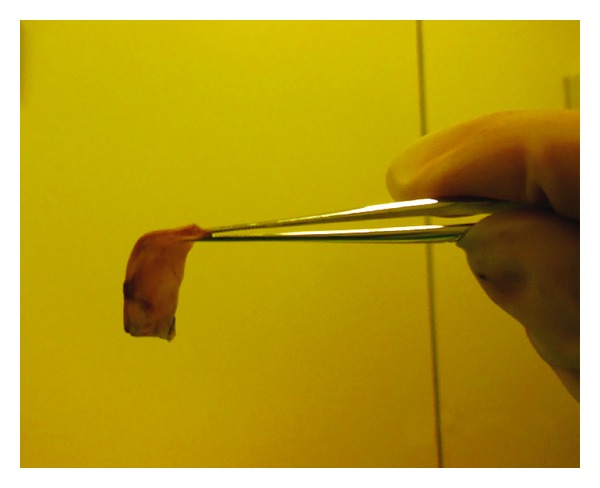
Scleral band photograph of the cross-linking procedure.

In treated eyes, a 10 mm × 20 mm scleral band was harvested sagittally from the 12 o'clock meridian (Figure 1), with the goal of obtaining equatorial and posterior sclera tissue. From each scleral band, two 3.5 mm × 15.0 mm scleral strips were dissected from the treated or corresponding control areas, avoiding muscle insertion points as much as possible. The tissue adjacent to the scleral strips was removed carefully. Scleral strip thickness was measured using micrometer calipers.

### 2.2. Cross-Linking Treatment

All scleral specimens were preserved in a 4°C moist chamber before treatment. The 0.1% riboflavin sodium phosphate was prepared by dissolving riboflavin sodium phosphate solution (Jiang'Xi pharmaceutical Co. Ltd., China) in distilled water. Fifteen minutes before the CXL treatment, 0.1% riboflavin solution was applied to the sclera every 3 min to facilitate deep scleral penetration of the riboflavin. The 0.1% riboflavin solution was also applied to the treatment area every 3 min during the 40 min irradiation. Scleral strips were irradiated with UVA (365 nm, UV-X 1000 system; IROC Innocross AG Co. Ltd., Switzerland) at an irradiance of 3 mW/cm^2^ and a distance of 5 cm from the scleral plane. Before each treatment, the desired surface irradiance was verified with a calibrated UVA meter at a distance of 5 cm. The treatment area was 9 mm × 9 mm in size and was located mainly in the posterior sclera.

### 2.3. Biomechanical Measurements

**Figure 2:**
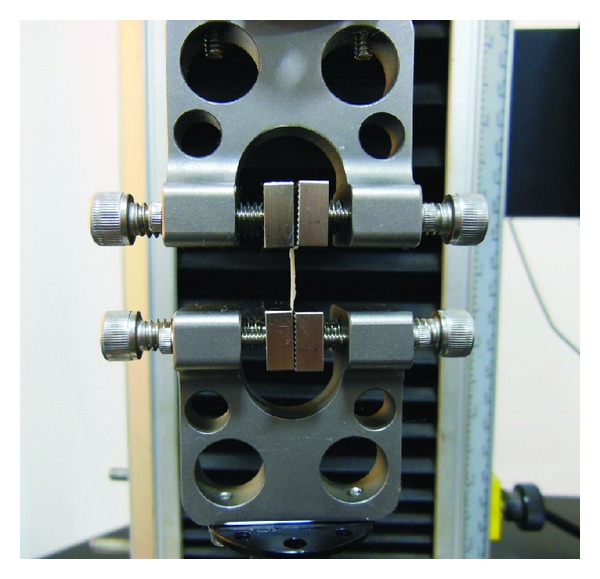
Evaluation of biomechanical properties of scleral strip was carried out on Instron 5544 system.

Stress-strain measurements were obtained for 48 scleral strips and 48 contralateral controls. The 3.5 mm × 15.0 mm scleral strips were clamped vertically between the jaws of the biomaterial tester (Instron 5544 system; Instron Co. Ltd., USA) with a distance of 4–6 mm between the clamps (Figure 2). In the treatment group, the tissue between the clamps was CXL tissue.

Specimens were loaded and unloaded under a constant velocity of 2 mm/min for 7 cycles to ensure accurate and consistent results. By the final cycle, the load displacement curves stabilized. The strain was then increased at a linear rate of 2 mm/min until the scleral specimen ruptured. The ultimate stress was measured as the stress on the tissue at the tearing point. Young's modulus, a measure of a tissue's elastic properties, was calculated as the slope of the stress-strain graph at 4%, 6%, and 8% strain.

### 2.4. Statistical Evaluation

Data of the ultimate stress, stress, and Young's modulus at a strain of 4%, 6%, and 8% were compared between the cross-linked treatment group and the untreated group using Student's *t*-tests. Statistical significance was defined as *P* ≤ 0.05.

## 3. Results

**Figure 3:**
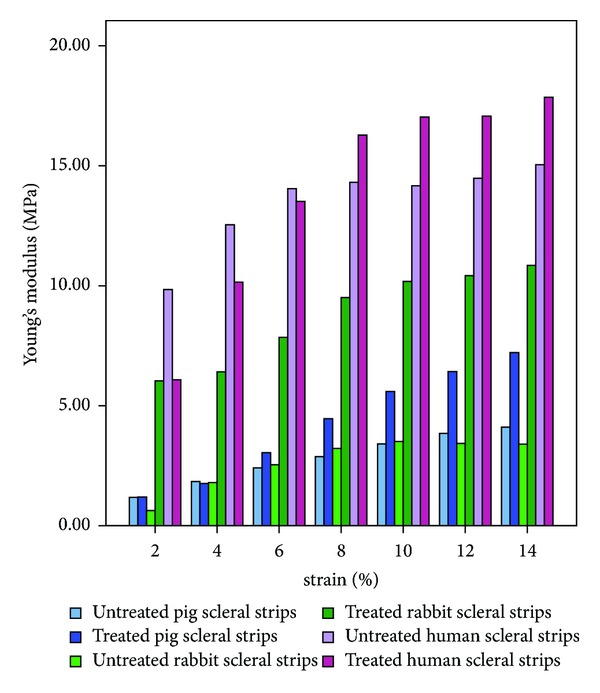
Young's modulus in porcine, rabbit, and human sclera.

Young's modulus at 8% strain was 2.88 ± 1.55 MPa, 4.46 ± 4.09 MPa, and 14.31 ± 8.56 MPa in untreated porcine, rabbit and human sclera, respectively. The stress was 0.2316 ± 0.1120 MPa, 0.3591 ± 0.3053 MPa, and 1.0788 ± 0.6458 MPa in untreated porcine, rabbit and human sclera, respectively. These data show that human sclera has a higher biomechanical stiffness than both porcine and rabbit sclera. Both stress and Young's modulus data demonstrate that human sclera is 4 times stiffer than porcine sclera and 3 times stiffer than rabbit sclera. Differences were particularly large in 4% and 6% strain measurements. Interestingly, the ultimate stress withstood by human sclera was lower than that withstood by porcine sclera (Table 1, Figure 3).

In rabbit sclera, both the stress and Young's modulus were significantly increased by riboflavin/UVA CXL (both *P* < 0.05). In porcine sclera, only the ultimate stress was significantly increased by CXL (*P* < 0.05). In human sclera, CXL did not significantly affect any of the biomechanical parameters (*P* > 0.05).

Scleral thickness of porcine, rabbit, and human eyes was 0.87 ± 0.18 mm, 0.30 ± 0.04 mm, and 0.41 ± 0.08 mm, respectively, in untreated strips and 0.96 ± 0.12 mm, 0.33 ± 0.04 mm, and 0.43 ± 0.09 mm, respectively, in treated strips. The CXL did not statistically affect sample thickness in any of the tissues examined (*P* > 0.05).

## 4. Discussion

The CXL induced by riboflavin/UVA can lead to a significant increase in biomechanical strength of both porcine and human sclera [6]. The treatment parameters in an earlier study were 3 mW/cm^2^ of 370 nm UVA light for 30 min. The apparatus consisted of 2 double UVA diodes (Roithner Lasertechnik), with overlapping irradiation fields for an irradiance of up to 6 mW/cm^2^ [6, 15]. In our experiment, the apparatus had no overlapping irradiation fields, and the irradiance energy was a uniform 3 mW/cm^2^. We believe that the lower amount of irradiation in our study compared to previous ones (3 mW/cm^2^ versus 6 mW/cm^2^) is the main reason that neither porcine nor human sclera had significant increases in biomechanical values.

Our study found a significant increase in scleral stiffness with riboflavin/UVA CXL in rabbit sclera, as demonstrated by an increase in Young's modulus by 113.2–264.2%, an increase in ultimate stress by 112.3%, and an increase in stress by 108.5–261.8%. In agreement with previous work [14, 15], this study found that riboflavin/UVA CXL is effective in altering the elastic properties of rabbit sclera. Interestingly, the same treatment did not change the biomechanical properties of human sclera and only had the effect of increasing porcine tissue ultimate stress by 42.1%.

We also found that the ultimate stress was raised significantly by CXL in porcine but not human tissue. These observations are in agreement with those of Wollensak and Spoerl [6], who found an increase in porcine sclera stress that was markedly higher than that in human scleral tissue with CXL by riboflavin/UVA (157% and 29%), glyceraldehyde (487% and 34%), and glutaraldehyde (817% and 122%). That is, the same method can induce different results in different species. This phenomenon could simply result from species-related variables in scleral structure but could also occur from differences in experimental design. Yang [16] believes that the elasticity modulus of soft tissue is affected by the ratio of collagen fibers and elastic fibers, with a higher proportion of collagen fibers correlating with a tissue higher firmness. Dias and Ziebarth [17] found that the elasticity of anterior cornea is higher than that of posterior cornea in a gradient pattern. Considering the varied constitution of fiber types in different layer of sclera and cornea, the effect of CXL on different layer of sclera should be studied to provide information for optimized CXL modality on sclera.

At 4%, 6%, and 8% strain, the stress in untreated porcine and rabbit sclera was similar to and lower than that in human sclera. The ultimate stress was higher in untreated porcine sclera than in rabbit sclera. The outcome of the riboflavin/UVA CXL of human sclera was similar to porcine sclera but not rabbit sclera.

Our previous study [18] has shown the enhancement of scleral stiffness in rabbit after CXL (>40 min duration); however, retinal damage was observed after more than 50 min of CXL. In order to obtain observable changes of scleral stiffness after CXL, as well as avoid possible injury of the retina, the present study was designed with an irradiation time of 40 min. Wang et al. [19] revealed that the stiffness of human sclera increases after CXL, as riboflavin instillation time increased from 5 to 20 min. Stiffness was maintained at a stable level when the instillation time was between 20 and 30 min. Although the riboflavin instillation duration was 15 min in present study, we plan to evaluate scleral biomechanical properties in different species following CXL with different riboflavin instillation duration.

Techniques to improve CXL are currently being sought. In dense, fibrous scleral tissue, the absorption of 365 nm UVA light is great and the transmission is negligible. Some studies found that because of differences in collagen fibril spacing and diameter, the light scattering, absorption, and transmission through the cornea was different in different areas of the cornea [20]. Doutch et al. [21] found that with 370 nm light, UV transmission decreases by about 20% from the corneal center to the periphery. In addition, riboflavin application alters the corneal UVA absorption coefficient [22, 23]. Therefore, ultrastructural differences among rabbit, porcine, and human sclera may result in differences in UVA absorption and transmission. This most likely means that different levels of UV-induced CXL take place in different tissue types. The smaller the UVA transmission, the less CXL occurs deep in the scleral tissue. This could explain why the same CXL treatment resulted in different tissue changes in rabbit, porcine, and human sclera. Because light transmission through the tissue is so important, varying the activation wavelength results in different sclera effects. Blue light, with a wavelength of 465 nm, has been utilized in sclera CXL with an impressive stiffening effect on rabbit tissue [7]. Further studies comparing the effect of blue light CXL on porcine and human sclera should be performed.

Previous experiments have demonstrated that rabbit and human sclera are permeable to compounds with a molecular weight up to 150 kDa [24, 25] and porcine sclera up to 120 kDa [26]. The photosensitizer riboflavin-5-phosphate has a molecular weight of 456 Da, which should easily penetrate the sclera. However, because the diffusion of riboflavin through the sclera is influenced by tissue structure, surface, thickness, and hydration [24, 27], riboflavin permeability between and within species can differ. In our study, the thicknesses of rabbit and porcine sclera were different and averaged 0.30 and 0.87 mm, respectively. These differences in permeability and thickness likely affected the results of our experiments because deeper scleral tissue CXL was almost certainly lessened by poor riboflavin infiltration [28].

All things considered, with the same irradiation dose (photosensitizer: 0.1% riboflavin drops, UVA: 3 mW/cm^2^, 365 nm, and 40 min), riboflavin/UVA CXL increases the biomechanical stiffness of rabbit sclera but not porcine or human sclera and compared with rabbit sclera, porcine sclera is closer to human sclera with respect to stress-strain biomechanical studies. Further research examining different energy doses and light wavelengths (e.g., blue light) is necessary to fully understand how to achieve optimum results with CXL procedures.

## Figures and Tables

**Table 1:** Stress values (MPa) for 4%, 6%, and 8% strain and calculated Young's modulus (MPa) in brackets.

Type of sclera	Stress at 4% (MPa)	Stress at 6% (MPa)	Stress at 8% (MPa)	Ultimate stress (MPa)
Porcine				
Untreated	0.0764 ± 0.0772 (1.95 ± 1.84)	0.1445 ± 0.0985 (2.41 ± 1.89)	0.2316 ± 0.1120 (2.88 ± 1.55)	5.06 ± 1.61
Treated	0.0754 ± 0.0419 (1.80 ± 1.05)	0.1581 ± 0.0779 (2.54 ± 1.30)	0.2533 ± 0.1120 (3.22 ± 1.46)	7.19 ± 2.22*
Rabbit				
Untreated	0.0725 ± 0.0729 (1.76 ± 2.03)	0.1848 ± 0.1680 (3.04 ± 3.03)	0.3591 ± 0.3053 (4.46 ± 4.09)	3.83 ± 2.87
Treated	0.2623 ± 0.1696* (6.41 ± 3.80)*	0.4985 ± 0.2928* (7.85 ± 4.34)*	0.7486 ± 0.4057* (9.51 ± 5.23)*	8.13 ± 3.99*
Human				
Untreated	0.4996 ± 0.3396 (12.54 ± 8.54)	0.8061 ± 0.4853 (14.05 ± 9.10)	1.0788 ± 0.6458 (14.31 ± 8.56)	4.45 ± 1.12
Treated	0.4112 ± 0.2988 (10.15 ± 7.54)	0.8039 ± 0.3997 (13.52 ± 7.77)	1.2592 ± 0.5817 (16.28 ± 7.33)	5.09 ± 1.88

*indicates a significant differences between the control and treated scleral strips (*P* < 0.05).
